# A Scoping Review of Arthropod‐Borne Flavivirus Infections in Solid Organ Transplant Recipients

**DOI:** 10.1111/tid.14400

**Published:** 2024-11-04

**Authors:** Seohyeon Im, Fadie Altuame, Isabel H. Gonzalez‐Bocco, Cilomar Martins de Oliveira Filho, Andrea Goldstein Shipper, Maricar Malinis, Carlo Foppiano Palacios

**Affiliations:** ^1^ Department of Internal Medicine Mass General Brigham‐Salem Hospital Salem Massachusetts USA; ^2^ Department of Neurology Brigham and Women's Hospital Boston Massachusetts USA; ^3^ Department of Neurology Massachusetts General Hospital Boston Massachusetts USA; ^4^ Division of Infectious Diseases Brigham and Women's Hospital Boston Massachusetts USA; ^5^ Department of Medical Oncology Dana‐Farber Cancer Institute Boston Massachusetts USA; ^6^ Cooper Medical School of Rowan University Camden New Jersey USA; ^7^ Department of Medicine Division of Infectious Diseases Vanderbilt University School of Medicine Nashville Tennessee USA; ^8^ Department of Medicine Division of Infectious Diseases Cooper Medical School of Rowan University Camden New Jersey USA

**Keywords:** arthropod‐borne flavivirus, dengue virus, solid organ transplant, West Nile virus

## Abstract

Arthropod‐borne flaviviruses (ABFs), transmitted by mosquitoes or ticks, are increasing due to climate change and globalization. This scoping review examines the epidemiology, clinical characteristics, diagnostics, treatment, and outcomes of ABF infection in solid organ transplant recipients (SOTRs). A database search up to January 25, 2024, focused on ABFs such as West Nile virus (WNV), dengue virus (DENV), Japanese encephalitis virus (JEV), Powassan virus (POWV), yellow fever virus (YFV), and Zika virus (ZIKV), limited to SOTRs.

We identified 173 WNV cases from 84 studies, with 28 donor‐derived infections (DDIs). Common clinical features included fever (78.5%), altered mental status (65.1%), and weakness or paralysis (45.6%). Treatment involved reducing immunosuppression (IS) in 93 cases, with intravenous immunoglobulin (IVIG), interferon alfa‐2b, and ribavirin used in 75 cases. Seven cases involved graft loss or rejection post‐infection. WNV infection had a 23.7% mortality rate, with severe neurological complications in 43.9%

For DENV infection, 386 cases from 47 studies were identified, including 14 DDI cases. Symptoms included fever (85%), myalgias (56.4%), and headache or retro‐orbital pain (34.6%). Severe dengue occurred in 50 cases (13.0%). IVIG was administered in six cases. Reduction in IS was reported in 116 patients. DENV mortality rate was 4.9%.

Additionally, 26 cases of less common ABFs such as JEV, POWV, YFV, and ZIKV were described.

In summary, ABF infections among SOTRs are associated with higher morbidity and mortality compared to the general population, emphasizing the need for improved preventive strategies, timely diagnosis, and optimized management protocols.

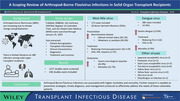

**Abbreviations**
ABF: arthropod‐borne flavivirus
ALT: alanine aminotransferase
AMS: altered mental status
AST: aspartate aminotransferase
ATG: anti‐thymocyte globulin
CNI: calcineurin inhibitor
CsA: cyclosporine
CSF: cerebrospinal fluid
DDI: donor‐derived infection
DENV: Dengue virus
DM: diabetes mellitus
IFN: interferon alfa‐2b
IHC: immunohistochemistry
IS: immunosuppression
IVIG: intravenous immunoglobulin
JEV: Japanese encephalitis virus
PCR: polymerase chain reaction
PLHIV: person living with human immunodeficiency virus
POWV: Powassan virus
PRNT: plaque reduction neutralization test
SLEV: Saint Louis encephalitis virus
SOT: solid organ transplant
SOTR: solid organ transplant recipient
TBEV: tick‐borne encephalitis virus
WHO: World Health Organization
WNND: West Nile neuroinvasive disease
WNV: West Nile virus
YFV: yellow fever virus
YFVV: yellow fever vaccine virus
ZIKV: Zika virus

## 1 Introduction

The genus Flavivirus comprises over 70 different single‐stranded, enveloped RNA viruses [1]. Most flaviviruses are arthropod‐borne, meaning they are transmitted to humans by hematophagous insects such as mosquitoes or ticks [2]. In recent decades, cases of arthropod‐borne flaviviruses (ABFs) have flared and spread to new geographical regions due to climate change and globalization [3, 4]. Some of the most notable flaviviruses with significant global endemic burden include West Nile virus (WNV), Dengue virus (DENV), Zika virus (ZIKV), Japanese encephalitis virus (JEV), and yellow fever virus (YFV).

In 2024, DENV cases surged across the Americas, surpassing 11 million cases by mid‐September. This is more than double the total cases reported in 2023 and has impacted 43 countries and territories [5]. Globally, Southeast Asia and South Asia bear the highest burden of DENV cases and deaths, while East Asia is experiencing the fastest rise in the age‐standardized incidence rate of DENV infections [6]. Although less common, other ABFs have the potential to emerge on a global scale. For instance, the Usutu virus garnered significant attention during the 2015–2016 outbreak with its spread being linked to fatalities across multiple European countries [7].

Solid organ transplant recipients (SOTRs) may be at increased risk of complications from ABF infections due to their need for immunosuppressive therapy. SOTRs may become infected with ABF if they reside in or have a travel history to endemic areas, receive blood transfusion with viral particles, or receive infected donor grafts [8]. Local transmission of DENV has recently been reported in the United States, posing a risk for SOTRs living in now endemic areas for DENV [9].

Despite increasing numbers of arboviral infections and solid organ transplants (SOTs) worldwide, there is limited literature on these endemic infections among SOTRs. This scoping review aims to identify the epidemiology, clinical characteristics, diagnostics, treatment, and outcomes of ABF infections among SOTRs to address existing knowledge gaps.

## 2 Methods

### 2.1 Search Strategy

To identify relevant studies for this scoping review, we conducted comprehensive searches in PubMed, EMBASE, the Cochrane CENTRAL Register of Controlled Trials (Wiley), and the Global Index Medicus (World Health Organization, WHO), including all studies published up to January 25, 2024. We used a combination of keywords and subject headings relevant to ABF infections in SOTRs. The search was developed and executed with the assistance of a medical librarian, ensuring adherence to the PRISMA‐S extension for search reporting [10]. We searched for studies on SOTRs with the following AFB: DENV, JEV, Saint Louis encephalitis virus (SLEV), tick‐borne encephalitis virus (TBEV), Powassan virus (POWV), WNV, YFV, ZIKV, Kunjin virus, Usutu virus, Omsk hemorrhagic fever virus, Murray Valley encephalitis virus, Kyasanur forest disease virus, and Alkhurma hemorrhagic fever virus. Detailed search strategies and a complete list of keywords can be found in [Supporting Information 1].

### 2.2 Data Selection

**FIGURE 1:**
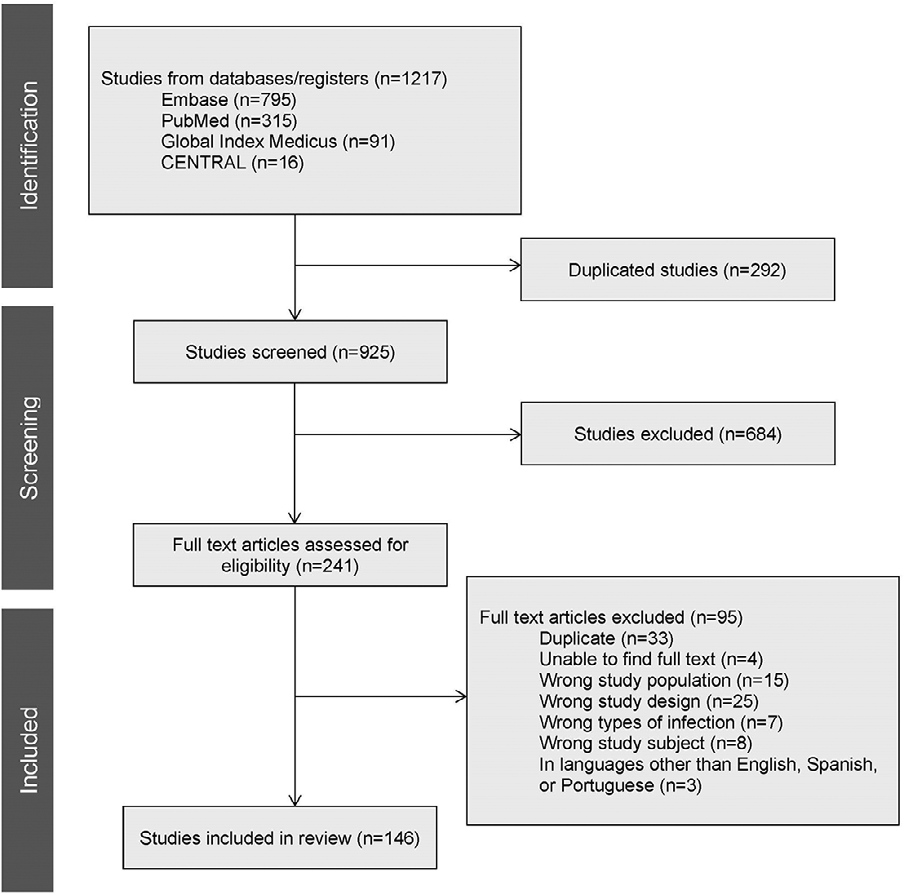
PRISMA 2020 flowchart.

The data selection process is illustrated in the PRISMA 2020 flowchart (Figure 1), which displays the screening process. Two independent reviewers screened the abstracts and titles of identified articles to determine their eligibility for full‐text review. In cases of disagreement, a third reviewer acted as a tiebreaker. The same process was applied during the full‐text screening phase.

We included published articles and abstracts of case reports, case series, prospective and retrospective cohort studies, and case‐control studies. Articles in English, Spanish, or Portuguese were included and interpreted by co‐authors proficient in these languages. Studies were excluded if they met the following criteria: incorrect study population (e.g., age < 18 years, no SOTR), incorrect study design (e.g., review article and guidelines), or incorrect types of infection (other than ABFs as listed above).

During full‐text review, studies deemed irrelevant—same exclusion criteria as abstract screening—were excluded. Additionally, studies of incorrect study subjects (e.g., serologic study, immunization prevalence, or in‐vitro‐only study) were excluded. Studies from the same country and research center were compared to ensure non‐overlapping research subjects or timeframes. If there is possible overlap, the less comprehensive study was excluded. Covidence (https://www.covidence.org/) was employed to facilitate the screening process. Detailed exclusion criteria and examples used during the screening process are provided in [Supporting Information 2].

### 2.3 Data Extraction

The following information was collected: patient demographics, transplant information (transplanted organ, donor status, induction therapy, maintenance immunosuppression [IS], rejection history, and time to infection onset), clinical characteristics (presentation, laboratory findings), diagnostic methods, treatment and outcomes (reduction in IS, treatment details, complications, rejection post‐infection, and death).

Definitions for laboratory findings include cerebrospinal fluid (CSF) lymphocytic pleocytosis (white blood cell count >5 /µL and more than 50% lymphocytes), CSF elevated protein (>45 mg/dL), thrombocytopenia (platelets <150 000/µL), and elevated aspartate aminotransferase (AST) or alanine aminotransferase (ALT) levels (AST >80 U/L or ALT >80 U/L; roughly 2 times the upper limit of normal [11]), anemia (Hemoglobin [Hb] <13 g/dL for men, <12 g/dL for women), neutropenia (absolute neutrophil count <1,500/µL), and lymphopenia (absolute lymphocyte count <1500/µL) Severe dengue was noted if manuscript described “severe dengue” or if symptoms or signs aligned with WHO 2009 guidelines: shock (described as “dengue shock syndrome,” “shock,” or hypotension needing vasopressors), severe bleeding (described as “severe bleeding,” acute bleeding needing red blood cell transfusion, or Hb <7), respiratory distress (respiratory distress due to fluid accumulation or needing advanced support (intubation or non‐invasive ventilation), elevated AST or ALT (either ≥1000 U/L), and severe organ involvement [12]. Data extraction details, including specific criteria, are described in [Supporting Information 3].

## 3 Results

The initial search using keywords yielded 1217 studies. After excluding 292 duplicate studies, 925 studies remained for the title and abstract screening. Of these, 241 studies were selected for full‐text screening. Ultimately, 146 studies on 585 SOTRs with reported ABF infections were included in the review.

Our review included 84 studies on WNV, 47 on DENV, two on JEV, two on POWV, two on SLEV, three on TBEV, three on YFV, and four on ZIKV among SOTRs. Among the YFV studies, we found two cases of yellow fever vaccine virus (YFVV). Additionally, there was a case of ZIKV and DENV co‐infection [13]. No publications were found on the Kunjin virus, Usutu virus, Omsk hemorrhagic fever virus, Murray Valley encephalitis virus, Kyasanur forest disease, or Alkhurma hemorrhagic fever virus infections among SOTRs.

### 3.1 West Nile Virus

#### 3.1.1 Patient Demographics

A total of 173 cases of WNV infection were identified from 84 studies [14, 15, 16, 17, 18, 19, 20, 21, 22, 23, 24, 25, 26, 27, 28, 29, 30, 31, 32, 33, 34, 35, 36, 37, 38, 39, 40, 41, 42, 43, 44, 45, 46, 47, 48, 49, 50, 51, 52, 53, 54, 55, 56, 57, 58, 59, 60, 61, 62, 63, 64, 65, 66, 67, 68, 69, 70, 71, 72, 73, 74, 75, 76, 77, 78, 79, 80, 81, 82, 83, 84, 85, 86, 87, 88, 89, 90, 91, 92, 93, 94, 95, 96, 97, 98]. The mean age of patients was 51.0 ± 14.3 years (*n* = 106). Among 144 patients with reported sex, 64.6% were male (93/144). Regarding comorbidities, one case included a person living with human immunodeficiency virus (PLHIV) [38] and 33 cases of diabetes mellitus (DM). Geographically, most cases were from North America, accounting for 91.3% (158/173) of cases, followed by Europe with 6.9% (12/173) and the Middle East with 1.7% (3/173). No studies reported cases from other regions. Most cases (99.4%, 171/172) were from WNV‐endemic areas, indicating exposure due to residence. Only one case was related to travel history to an endemic area, specifically Friuli‐Venezia Giulia in northeast Italy, known as a WNV‐affected area [60] (Table 1).

**TABLE 1:** Demographics and transplant information in West Nile virus (WNV) and Dengue virus (DENV) infections.

	West Nile virus	Dengue virus
	*n* = 173	*n* = 386
	No. (%)	
**Patient demographics**		
Sex	144 (100)	382 (100)
Male	93 (64.6)	246 (64.4)
Female	51 (35.4)	136 (35.6)
Comorbidities	34 (100)	12 (100)
HIV	1 (2.9)	0
DM	33 (97.1)	12 (100)
Region	173 (100)	386 (100)
North America	158 (91.3)	0
Europe	12 (6.9)	0
Middle East	3 (1.7)	0
South/Central America	0	146 (37.8)
South Asia	0	169 (43.8)
Southeast Asia	0	57 (14.8)
East Asia	0	5 (1.3)
Africa	0	8 (2.1)
Oceania	0	1 (0.3)
Endemicity exposure, *n*	173 (100)	386 (100)
Residence	172 (99.4)	384 (99.5)
Travel history	1 (0.6)	2 (0.5)
**Transplant information, IS**		
Transplanted organ	167 (100)	386 (100)
Kidney	91 (54.5)	369 (95.6)
Liver	25 (15.0)	16 (4.1)
Heart	22 (13.2)	1 (0.3)
kidney/pancreas	14 (8.4)	0
Lung	8 (4.8)	0
Pancreas	3 (1.8)	0
Intestine	1 (0.6)	0
Others	3^a^ (1.8)	0
Donor	72 (100)	260 (100)
Deceased	56 (77.8)	127 (48.8)
Living	16 (22.2)	133 (52.2)
Induction therapy	37 (100)	87 (100)
ATG	22 (59.5)	47 (54.0)
Basiliximab	10 (27.0)	41 (4.1)
Alemtuzumab	4 (10.8)	0
Muromonab	1 (2.7)	0
Maintenance IS	128 (100)	282 (100)
CNI	124 (96.9)	248 (87.9)
Mycophenolate	101 (78.9)	216 (76.6)
Steroids	83 (64.8)	265 (94.0)
Azathioprine	2 (1.6)	35 (12.4)
Sirolimus	5 (3.9)	24 (8.5)
Rejection history prior to infection	19/173 (11.0)	15/386 (3.9)
Onset since SOT, interval	103 (100)	129 (100)
<30 d	37 (35.9)	34 (26.4)
30 d ≤ ≤ 1 y	31 (30.1)	17 (13.2)
>1 y	35 (34.0)	78 (60.5)
Transmitted from organ	28/173 (16.2)	14/173 (3.7)
Transmitted from transfusion	6/173 (3.5)	0

Abbreviations: ATG, Antithymocyte globulin; CNI, calcineurin inhibitor; d, day; DM, diabetes mellitus; HIV, human immunodeficiency virus; IS, immunosuppression; m, month; SOT, solid organ transplant; y, year.

^a^ Kidney/heart (*n* = 2), kidney/liver (*n* = 1).

#### 3.1.2 Transplant Information and Immunosuppression

The most common transplanted organ was the kidney (54.5% 91/167), followed by the liver (15.0%, 25/166) and heart (13.2%, 22/166). Among 72 cases reporting donor status, 77.8% (56/72) were from deceased donors. Induction therapy was mentioned in 37 reported cases; the most commonly used agents were anti‐thymocyte globulin (ATG) at 59.5% (22/37), basiliximab at 27.0% (10/37), and alemtuzumab at 10.8% (4/37). 127 cases reported maintenance IS at the onset of infection. The most common maintenance immunosuppressives included calcineurin inhibitors (CNI) with 96.9% (123/127), mycophenolate with 78.7% (100/127), and corticosteroids with 64.6% (82/127). Graft rejection prior to WNV infection was reported in 19 cases [24, 29, 35, 42, 43, 63, 71, 82]. Four WNV cases occurred within 6 months after rejection, which had been treated with pulse steroids, intravenous immunoglobulin (IVIG), and rituximab. One WNV case occurred within 1 year after rejection, which had been treated with ATG. Eleven cases reported graft rejection over 6 months before WNV infection, and the remaining cases did not report the timing of rejection prior to WNV infection. Time to infection onset from SOT was available for 103 cases. Of these, 35.9% (37/103) occurred within 30 days, 30.1% (31/103) occurred between 30 days and 1 year, and 34.0% (35/103) more than 1 year post‐SOT. Donor‐derived infection (DDI) was reported in 28 cases [14, 26, 27, 30, 32, 36, 42, 45, 61, 68, 89, 91, 92, 96], and transmission through transfusion of blood products was reported in six cases [17, 44, 48, 49, 69, 82] (Table 1).

#### 3.1.3 Clinical Characteristics

Clinical presentation was reported in 149 cases. Clinical manifestations included fever in 78.5% (117/149), altered mental status (AMS) in 65.1% (97/149), weakness or paralysis in 45.6% (68/149), headache in 30.2% (45/149), and seizures in 13.4% (20/149). Three cases reported asymptomatic infections in the setting of donor‐derived transmission (Table 2).

**TABLE 2:** Clinical characteristics and outcomes of West Nile virus (WNV) infections.

	West Nile virus
	*n* = 173
	No. (%)
**Clinical characteristics**	
Presentation	149 (100)
Fever	117 (78.5)
AMS	97 (65.1)
Weakness/paralysis	68 (45.6)
Headache	45 (30.2)
Diarrhea	25 (16.8)
Seizures	20 (13.4)
Neck stiffness	7 (4.7)
Asymptomatic	3^a^ (2.0)
**Diagnosis**	
Non‐radiologic diagnostics	150 (100)
Serum IgM	90 (60.0)
Serum IgG	50 (33.3)
PRNT	13 (8.7)
Serum PCR	26 (17.3)
CSF lymphopleocytosis	62 (41.3)
CSF elevated protein	66 (44.0)
CSF IgM	46 (30.7)
CSF IgG	17 (11.3)
CSF PCR	27 (18.0)
Autopsy	15 (10.0)
Brain biopsy	2 (1.3)
MRI Brain	73 (100)
Signal abnormalities	48 (65.8)
Normal/nonspecific	25 (34.2)
**Treatment/outcomes**	
IS reduction	93/173 (53.8)
Treatment	75 (100)
IVIG	69 (92.0)
IFN	16 (21.3)
Ribavirin	4 (5.3)
Others	4^b^ (5.3)
Non‐fatal sequelae	66 (100)
Mild/asymptomatic	37 (56.1)
Severe NC	29 (43.9)
Coma	6 (9.1)
Paralysis	15 (22.7)
Discharged to LTAC	3 (4.5)
non‐specified	5 (7.6)
Graft loss/rejection	7/173 (4.0)
Related death	41/173
Attributable mortality	23.7%

Abbreviations: AMS, altered mental status; CSF, cerebrospinal fluid; IFN, interferon; IVIG, intravenous immunoglobulin; LTAC, long‐term acute care.; MRI, magnetic resonance imaging; NC, neurological complication; PCR, polymerase chain reaction; PRNT, plaque reduction neutralization test.

^a^ All cases were from donor‐derived infection.

^b^ Plasmapheresis (*n* = 1), WNV‐Ab‐enriched FFP (*n* = 2), placebo‐controlled double‐blind study comparing IVIG or saline (*n* = 1).

#### 3.1.4 Diagnosis

Non‐radiologic diagnostics for WNV infection were reported in 150 cases [Table 2]. Serum IgM was positive in 60.0% (90/150) of cases, serum IgG in 33.3% (50/150), plaque reduction neutralization tests (PRNTs) in 8.7% (13/150), and serum polymerase chain reaction (PCR) in 17.3% (26/150). CSF studies identified elevated protein levels in 44.0% (66/150) and lymphocytic pleocytosis in 41.3% (62/150). CSF IgM was positive in 30.7% (46/150), CSF IgG in 11.3% (17/150), and CSF PCR in 18.0% (27/150). Brain biopsy was described in two cases. One case showed findings consistent with encephalitis in the right caudate nucleus, while the other revealed inflammatory infiltrate, necrosis, and vacuolation, with positive WNV PCR from the biopsy tissue [49, 87]. Autopsies were performed in 15 cases; brain tissue samples revealed edema, hemorrhagic necrosis, microscopic findings consistent with encephalomeningitis, positive immunohistochemistry (IHC) for WNV or flavivirus antigens, and WNV RNA PCR [20, 27, 30, 32, 33, 35, 39, 40, 48, 54, 58, 61, 73, 77]. One autopsy study found WNV antigens by IHC in multiple organs including the kidney, lung, and pancreas [58]. Regarding radiologic findings, all 32 computed tomography head reports were described as normal or nonspecific. 73 magnetic resonance imaging (MRI) scan results were available, and 65.8% (48/73) demonstrated abnormal MRI findings (Table 2).

#### 3.1.5 Treatment and Outcomes

Reduction in IS was reported in 93 cases. Treatment other than supportive management was reported in 75 cases. IVIG was used in 92.0% (69/75), interferon alfa‐2b (IFN) in 21.3% (16/75), and ribavirin in 5.3% (4/75). Other measures were used in three cases, including WNV antibody‐enriched fresh frozen plasma (*n* = 2), and plasmapheresis (*n* = 1). Among 66 cases reporting non‐fatal outcomes, severe neurological complications accounted for 43.9% (29/66): 9.0% (6/66) experienced coma, 22.7% (15/66) experienced paralysis, and 4.5% (3/66) were discharged to long‐term acute care facilities. Additionally, 7.5% (5/66) cases had non‐specified severe neurological deficits, including three patients reported as having “severe neurologic damage” [29, 82]; one patient with an overall decreased functional independence score requiring 6 weeks of inpatient rehabilitation, tracheostomy, and gastrostomy tube [16]; and one patient with impaired arousal, ataxic dysarthria, aphasia, persistent vertical nystagmus, and motor impairments [71]. Conversely, 56% (37/66) exhibited mild or non‐specific complications, such as headache, dizziness, and partial motor deficits like foot drop. Seven cases had graft loss or rejection post‐infection [24, 27, 29, 40]. One study found three patients suffered acute rejection within 1 year of infection, and two patients lost allografts after more than 1 year from infection [29]. The timeline of graft loss or rejection in other cases was unclear. Death was common post‐WNV infection in 41 post‐SOT cases, resulting in an attributable mortality rate of 23.7% (Table 2).

### 3.2 Dengue Virus

#### 3.2.1 Patient Demographics

386 patients with DENV infection were identified from 47 studies [13, 97, 99, 100, 101, 102, 103, 104, 105, 106, 107, 108, 109, 110, 111, 112, 113, 114, 115, 116, 117, 118, 119, 120, 121, 122, 123, 124, 125, 126, 127, 128, 129, 130, 131, 132, 133, 134, 135, 136, 137, 138, 139, 140, 141, 142, 143]. The mean age of patients was 45.4 ± 14.4 years (n = 107). Among 382 patients with reported sex, 64.4% were male (246/382). Regarding comorbidities, 12 cases of DM were reported and no PLHIV was noted. Most cases were from South Asia (43.8%, 169/386), followed by South/Central America (37.8%,146/386), and Southeast Asia (14.8%, 57/386). Most cases (99.5%, 384/386) were from DENV‐endemic areas, indicating local exposure. Travel history to endemic areas was reported in only two cases (0.5%, 2/386), involving travel to Samoa and Thailand [13, 99] (Table 1).

#### 3.2.2 Transplant Information and Immunosuppression

Most transplanted organs were kidneys (95.6%, 369/386), followed by livers (4.1%, 16/386) and hearts (0.3%, 1/386). Among 260 cases reporting donor status, 127 (48.8%) were from deceased donors.

87 reported cases reported induction immunosuppressive therapy: ATG (54.0%, 47/87) and basiliximab (47.1%, 41/87) only. No cases received alemtuzumab. Maintenance IS at the onset of infection was reported in 282 cases. The most common immunosuppressives included corticosteroids (94.0%, 265/282), CNI (87.9%, 248/282), and mycophenolate (76.6%, 216/282).

Graft rejection prior to DENV infection was reported in 15 cases, but all cases reported prior graft rejection more than 6 months before DENV infection [102, 105, 107, 111, 120, 129]. Time to infection onset from SOT was available for 129 cases. Of these, 26.4% (34/129) occurred within 30 days, 13.2% (17/129) occurred between 30 days and 1 year, and 60.5% (78/129) more than 1 year post‐SOT. Donor‐derived DENV infection was reported in 14 cases [100, 101, 125, 127, 131, 132, 136, 141, 142]. No transfusion‐derived cases were identified (Table 1).

#### 3.2.3 Clinical Characteristics

Clinical presentation was reported in 381 cases. The most common symptom was fever (85%, 324/381), myalgias (56.4%, 215/381), and headache or retro‐orbital pain (34.6%, 132/381). Two asymptomatic cases were reported in the setting of donor‐derived transmission. Among 280 cases with laboratory values, the most common findings included thrombocytopenia (81.8%, 229/280), leukopenia (47.1%, 132/280), and elevated AST or ALT levels (41.4%, 116/280). According to the 2009 WHO classification, severe dengue was reported in 50 cases (13.0%, 50/386). Among these, 46.0% (23/50) experienced shock, 28.0% (14/50) had severe bleeding, 16.0% (8/50) had respiratory failure, and 6.0% (3/50) had AST or ALT levels ≥1000 U/L. Additionally, there were 10 cases of organ failure, which included multi‐organ failure, acute pancreatitis or acute kidney injury, and two cases of encephalitis [Table 3].

**TABLE 3:** Clinical characteristics and outcomes of Dengue virus (DENV) infections.

	Dengue virus
	*n* = 386
	No. (%)
**Clinical characteristics**	
Presentation	381 (100)
Fever	324 (85.0)
Myalgia	215 (56.4)
HA/Retro‐orbital pain	132 (34.6)
Nausea/vomiting	63 (16.5)
Arthralgia	52 (13.6)
Diarrhea	58 (15.2)
Bleed/hemorrhage	51 (13.4)
Abdominal pain	45 (11.8)
Rash	17 (4.5)
Asymptomatic	2^a^ (0.2)
Laboratory values	280 (100)
Thrombocytopenia	229 (81.8)
Leukopenia	132 (47.1)
Elevated AST/ALT	116 (41.4)
Anemia	25 (8.9)
Neutropenia	12 (4.3)
Lymphopenia	1 (0.4)
Severe Dengue	50 (100)
Shock	23 (46.0)
Severe bleeding	14 (28.0)
Respiratory failure	8 (16.0)
AST or ALT ≥1000	3 (6.0)
Other severe features	12^c^ (24.0)
**Diagnosis**	
Diagnostic method	255 (100)
NS1 Antigen	136 (53.3)
Serum IgM	136 (53.3)
Serum IgG	26 (10.2)
Serum PCR	64 (25.1)
Others	6^b^ (2.4)
**Treatment/outcomes**	
IS reduction	116/386 (30.1)
Treatment	6 (100)
IVIG	6 (100)
Graft loss or rejection	5 (1.3%)
Related death	19/386
Attributable mortality	4.9%

Abbreviations: ALT, alanine aminotransferase; AST, aspartate aminotransferase; HA, headache; IS, immunosuppression.; IVIG, intravenous immunoglobulin; PCR, polymerase chain reaction.

^a^ All cases were from donor‐derived infection.

^b^ Unspecified ELISA (*n* = 1), PCR and pathology from liver (*n* = 2), urine PCR (*n* = 1), CSF IgG (*n* = 1).

^c^ Encephalitis (*n* = 2), organ failure (*n* = 10).

#### 3.2.4 Diagnosis

Diagnostic methods were reported in 255 cases. Positive diagnostic tests included NS1 antigen in 53.3% (136/255), serum IgM in 53.3% (136/255), serum PCR in 25.1% (64/255), and serum IgG in 10.2% (26/255) (Table 3).

#### 3.2.5 Treatment and Outcomes

116 cases reported reducing IS. Six patients received IVIG for treatment of DENV infection. Five cases reported graft loss or rejection post‐infection with unclear timelines. There were 19 DENV‐related deaths, resulting in an attributable mortality rate of 4.9% (Table 3).

### 3.3 Other ABFs

A total of 26 cases of JEV, POWV, SLEV, TBEV, YFV, YFVV, and ZIKV infections were identified from 15 studies [13, 144, 145, 146, 147, 148, 149, 150, 151, 152, 153, 154, 155, 156, 157] (Table 4).

**TABLE 4:** Summary of other arthropod‐borne flaviviruses (ABFs).

Virus	Study	Country	Cases	Age, mean	Transplanted organ
JEV	Cheng 2018 [144]	China	1	52	Lung
	Qi 2020 [145]	China	1	67	Liver
Powassan	Destrampe 2019 [146]	US	1	38	Kidney
	Taylor 2021 [147]	US	1	30	NR
SLEV	Hartmann 2017 [148]	US	3	64.3	Kidney (*n* = 2), kidney‐heart (*n* = 1)
TBEV	van den Bogaart 2022 [149]	Switzerland	2	NR	NR
	Veje 2014 [150]	Sweden	1	70	Kidney
	Lipowski 2017 [151]	Poland	3	43	Kidney (*n* = 2), liver (*n* = 1)
YFV	Pierrotti 2020 [152]	Brazil	1	58	Kidney
YFVV	de Sousa 2019 [153]	Brazil	1	50	Kidney
	Gould 2023 [154]	US	4	47.5	Kidney (*n* = 2), liver (*n* = 1), heart (*n* = 1)
Zika	Schwartzmann 2017 [155]	Brazil	1	36	Heart
	Dos Santos 2016 [156]	Brazil	1	55	Liver
	Nogueira 2017 [157]	Brazil	4	56	Kidney (*n* = 2), liver (*n* = 2)
Zika/Dengue	Chan 2016 [13]	Australia	1	63	Kidney

Abbreviations: JEV, Japanese encephalitis virus; NR, not reported; SLEV, Saint Louis encephalitis virus; TBEV, Tick‐borne encephalitis virus; US, United States; YFV, yellow fever virus; YFVV, yellow fever vaccine virus.

#### 3.3.1 Japanese Encephalitis Virus

JEV after SOT was reported in two cases in the literature [144, 145]. A 52‐year‐old male from Hong Kong with advanced chronic obstructive pulmonary disease underwent a double‐lung transplant and received a blood transfusion from an asymptomatic viremic donor 43 days after transplantation [144]. 14 days after the blood transfusion, the patient developed fever and maculopapular rash; over the course of 2 days, symptoms progressed to confusion and myoclonus. Another case was a 67‐year‐old female from China with autoimmune cirrhosis who underwent orthotopic liver transplantation [145]. The patient developed fever and AMS 13 days after transplant and DDI could not be ruled out. The diagnosis was confirmed by positive serum and CSF serologies in both cases and positive CSF PCR for JEV in one case [144, 145]. One patient was treated with IVIG [145]. Another patient died 3 months after initial symptoms, unclear if related to JEV; and another patient fully recovered without neurological sequelae [144, 145].

#### 3.3.2 Powassan Virus

There were only two published reports on POWV infections following blood transfusions in SOTRs [146, 147]. In one case, a 38‐year‐old cadaveric renal transplant recipient developed POWV encephalitis 3 weeks post‐transplant [146]. Another case was a 30‐year‐old female who received a live donor kidney and initially presented with headache, fever, weakness, myalgia, and diarrhea within 24 hours post‐transplant [147]. Her condition progressed to tremors, ataxia, dysarthria, sensorineural hearing loss, and bilateral blurred vision. Both patients had no known tick exposure, banked serum donor samples tested negative for POWV, and were both confirmed to have red blood cell transfusion‐associated POWV transmission [146, 147]. Diagnosis, management, and outcome for the first case were not reported [146]. The second case underwent an MRI that showed pial enhancement in the cerebellum, consistent with cerebellitis [147]. She was diagnosed with positive serum and CSF IgM and serum PRNT (highest titer 1:320); while CSF PRNT and serum and CSF PCR were negative. Initially, the patient was given corticosteroids due to a concern for autoimmune encephalitis. Five months after discharge, the patient fully recovered, and MRI cerebellar enhancement resolved.

#### 3.3.3 Saint Louis Encephalitis Virus

Three cases of SLEV after SOT were found in a single case series from Phoenix, Arizona [148]. This case series included three unrelated male recipients, including one kidney‐heart and two kidney recipients. One patient developed symptoms within 5 weeks of kidney transplantation in the setting of blood transfusion 21 days after transplant, and the other two cases occurred several years after transplantation. Symptoms included fever, rigors, diarrhea, headache, and confusion. Diagnosis was confirmed in all patients by positive CSF SLEV serological and PRNT testing. One patient also had positive serum SLEV serological and PRNT testing done at the Centers for Disease Control and Prevention. Treatment for two patients in the case series included IFN and IVIG. One patient died within 3 days of hospitalization; another had dysarthria as the only sequelae and the last patient recovered without further neurological sequelae.

#### 3.3.4 Tick‐Borne Encephalitis Virus

Three reports described TBEV after SOT [149, 150, 151]. The Swiss Transplant Cohort Study reported two cases of TBEV infection in a cohort study on CNS infections but provided no further details [149]. In another case, a 70‐year‐old kidney transplant patient from Sweden developed weakness in the lower extremities and hands [150]. CSF testing showed lymphocytic pleocytosis and serum IgM was positive for TBEV, although IgG and PCR testing were negative. Urine TBEV PCR was positive. The patient experienced progressive AMS and eventually died. A case series described donor transmission of TBEV from a donor in northern Poland to one liver and two kidney recipients [151]. These patients presented 17–49 days post‐transplantation with fever, headache, vertigo, emesis, cranial nerve palsies, and aphasia. Only one patient had CSF pleocytosis and elevated protein. All patients were diagnosed with encephalitis that progressed to coma, and all subsequently died. Donor brain tissue tested positive for TBEV by PCR. One patient's CSF was positive for TBEV by PCR and next‐generation sequencing, and two brain tissue samples were positive for TBEV by PCR and next‐generation sequencing.

#### 3.3.5 Yellow Fever Virus

YFV infection after SOT was described in one published report [152]. A 58‐year‐old male, 24 years post‐transplant, traveled to a YFV‐endemic area in Brazil and developed a high fever, rapid deterioration to shock, fulminant hepatitis, coma, and eventually death. Serum YFV PCR was positive, and autopsy revealed YFV antigens in the liver, spleen, and brain.

Two publications reported outcomes of YFVV infection among SOTRs [153, 154]. During the 2018 YFV outbreak in Brazil, a kidney transplant recipient, 5 years post‐transplant, received a reduced dose of YFV vaccination against medical advice [153]. The patient developed nausea, fever, myalgias, arthralgias, and diarrhea, with thrombocytopenia and elevated AST or ALT levels, and was found to have positive blood YFV PCR. The patient experienced antibody‐mediated rejection 1 month later but recovered without further issues. In the US case series, a deceased organ donor had received a blood transfusion from a donor recently vaccinated against YFV [154]. The liver, heart, and two kidney recipients developed symptoms including fever, headache, nausea, emesis, diarrhea, AMS, and left hemiparesis 15–40 days post‐transplant. The diagnosis was confirmed by positive YFV CSF IgM in three patients, positive YFV serum IgM in three patients, and metagenomic next‐generation CSF sequencing in one patient. An autopsy on one patient revealed YFV RNA in brain tissue by PCR. One patient had reduced IS, and two received IVIG. Two patients died, while the other two survived without neurologic sequelae. The right cornea recipient developed headaches 40 days post‐transplant but tested negative for YFV by IHC and PCR and underwent corneal transplant replacement. The left cornea recipient remained asymptomatic.

#### 3.3.6 Zika Virus

Two case reports and one case series described ZIKV infection in SOTRs [155, 156, 157]. In one case report, a 36‐year‐old heart transplant recipient, 8 months post‐transplant, developed fever, headache, and seizures [155]. Notably, the patient's family members experienced fever and rash. CSF analysis revealed lymphocytic pleocytosis, elevated protein levels, and positive ZIKV PCR. The patient died from cardiogenic shock secondary to acute cardiac allograft rejection. Another case involved a liver transplant recipient who received a platelet transfusion from a donor with asymptomatic ZIKV infection [156]. The patient's only symptom was persistent thrombocytopenia post‐transplantation. Platelet donors subsequently developed “dengue‐like symptoms”. Both donor and recipient tested positive for ZIKV by PCR, and viral genomic sequencing confirmed autochthonous transmission. A case series included two kidney and two liver transplant recipients, with cases occurring 43–590 days post‐transplant [157]. Patients presented with fever, headache, myalgia, vomiting, anemia, thrombocytopenia, and organ failure. Positive blood RT‐PCR confirmed ZIKV infection in all cases. All patients received supportive care, with no deaths or long‐term sequelae.

One case report described co‐infection with ZIKV and DENV in a 63‐year‐old renal transplant recipient, 6 years post‐transplant and on cyclosporine (CsA) and prednisolone [13]. After recent travel to Samoa, the patient presented with malaise, thrombocytopenia, hyperbilirubinemia, and elevated liver enzymes (ALT 1500 U/L; AST 3000 U/L). Blood PCR was positive for both ZIKV and Dengue serotype 3. Patient made a full recovery.

## 4 Discussion

We conducted a scoping review of ABF infections in SOTRs, including a review of 146 studies. Most infections occurred in patients living in endemic areas for ABF, rather than through exposure by travel to endemic areas. Common symptoms across all viruses included fever, headache, and nonspecific gastrointestinal symptoms. Neurological abnormalities were reported with WNV, JEV, TBEV, and POWV. The mainstay of treatment for most cases included a reduction in iatrogenic IS, but antiviral treatments and immunoglobulins were used in a subset of cases, with varying outcomes. We found that ABF infections can lead to significant morbidity and mortality among SOTRs. This scoping review is the first to comprehensively analyze the available literature on clinical manifestations, diagnostics, treatment strategies, and outcomes of ABF infections among SOTRs.

Most ABF studies among SOTRs in the literature reported WNV infection. Most WNV infections originated in North America, with additional cases reported in Europe and the Middle East, primarily affecting patients living in endemic regions. We found that WNV infection can occur at any time after SOT, due to donor‐derived transmission, blood product transfusion, or de‐novo infection. Transmission of WNV via blood and organ donation was reported in 34 cases among SOTRs, including case series of multiple‐donor‐derived infections from common donors [30, 45, 61, 89, 91]. Notably, in a case series involving four SOTRs infected with WNV from a common donor, PCR testing of the donor's stored serum at the time of donation was negative for WNV. However, PCR of the donor's lymph nodes and spleen tested positive for WNV RNA, suggesting potential sources of transmission from non‐solid organ or hematopoietic tissues [27]. Recent guidelines recommend WNV screening for living donors in endemic areas during times of increased WNV transmission; however, there are no established recommendations for screening deceased donors [158]. In our study, all (17 cases) donor‐derived WNV infection with reported donor status were from deceased donors, further highlighting the need for consensus on WNV screening of deceased donors.

In the general population, only 25% of WNV infections develop fever, and less than 1% progress to West Nile neuroinvasive disease (WNND) [159]. Reports on the presentation of WNV infection among SOTRs have been conflicting [160, 161, 162, 163]. One seroprevalence study among SOTRs following the WNV epidemic found that the risk of developing WNND was 40% among patients with WNV infection [161]. However, a national surveillance study from the US and another retrospective study found no significant association between SOT and WNND [160, 163]. Despite these conflicting reports, our findings indicate that SOTRs are commonly present with AMS, paresis, or seizures. This is consistent with a recent retrospective cohort study which demonstrated higher rates of neurological symptoms in immunocompromised compared to immunocompetent adults with WNND [164].

Diagnostic methods varied for WNV: most cases used serology from serum or CSF, and few cases (<20%) used PCR; consistent with the known short viremic phase of WNV and the need for serological testing [165]. Given the immunocompromised state of SOTRs, delayed seropositivity and false‐negative serology can occur. Hence, the a need for multiple diagnostic modalities to confirm diagnosis [25, 166]. Prolonged positive WNV PCR, from hospital day 5 (serum) until autopsy of the brain after 99 days, has been documented previously in a patient who was diagnosed with B cell lymphoma and received chemotherapy [167], but the difference in length of viremia between immunocompetent and immunocompromised populations is unknown.

All patients received supportive treatment, and explicit reduction in IS was documented in 93 cases. The primary treatment for WNV infection is supportive care, and reducing IS is crucial to restoring natural immunity [158]. We found several cases of adjunctive treatment with IVIG, IFN, and ribavirin. IVIG treatment, including high titer WNV‐specific immunoglobulin (Omr‐IgG‐am) and standard IVIG, showed no significant efficacy in one US‐based randomized clinical trial but can be theoretically beneficial for individuals with humoral deficiencies [158, 168]. Current guidelines state that IVIG administration for WNV infections among SOTRs can be considered, with weak recommendations with moderate evidence [158]. IFN treatment for WNV infection had variable outcomes, but there is concern about IFN treatment in SOTRs due to the potential risk of graft rejection [158]. Ribavirin has demonstrated in vitro activity against WNV infection but has no proven clinical efficacy [158, 169].

Regarding mortality and sequelae post‐WNV infection in SOTRs, our review found high mortality (23.7%) and prevalence of severe neurological outcomes (43.9%). This is higher than the general population; in the US, mortality of WNV infection was 5.0% (from 1999 to 2023, 2958 deaths from 59.151 cases [170]). Additionally, a prospective cohort study for WNV infection and neurological recovery showed persistent mild symptoms in 47% in 2 years post‐onset, but only 4% had paralysis, and the rest of the symptoms correlate to mild neurological complications [171]. One systematic review of WNV infection in SOTRs reported 36.54% mortality and long‐term neurological sequelae in 18.75% [43]. High morbidity and mortality in WNV infection SOTRs further emphasize the need to prevent disease transmission and improve treatment regimens for SOTRs.

DENV infection was the most common ABF infection (386 cases). Most DENV infections were predominantly from Southeast Asia and South America, particularly among patients residing in endemic areas. Timing of DENV infection post‐transplantation varied but was more common after the first year from SOT (60.5%). However, 39.5% of cases occurred within the first years after SOT, suggesting closer monitoring and proactive management strategies for SOTRs with endemic exposure. Transmission of DENV via organ donation was reported in 14 recipients [100, 101, 125, 127, 131, 132, 136, 141, 142]. To prevent donor transmission, South Asian transplant guidelines suggest screening deceased and living donors during peak DENV season [172]. Brazilian transplant guideline recommends screening living and deceased donors and recipients without a clinical picture of dengue using serum NS1 and IgM [173]. Given that DENV is now endemic in certain regions of the US, transplant centers in endemic areas should consider screening donors to prevent DDI and testing symptomatic SOTRs living in endemic areas for DENV [9].

Common symptoms among SOTRs with DENV infection included fever, myalgia, and headache or retro‐orbital pain, similar to symptoms in the general population during the febrile phase [174].

We found that IgM and NS1 antigen testing were used equally to diagnose DENV. Centers for Disease Control and Prevention recommend testing for DENV PCR and IgM or NS1 antigen and IgM testing within the first 7 days of symptoms, and IgM testing only after 7 days [175]. Both assays are recommended and may benefit from enhanced diagnostic performance when used in combination [176]. NS1 detection is the test of choice during the febrile phase, as it can appear before day 5 [176]. DENV PCR testing can also be performed prior to day 7 of symptoms. DENV IgM detection peaks on days 10–14 and disappears after 3 months [176]. However, recent infections with related flaviviruses, such as YFV or ZIKV, may lead to false‐positive IgM results [177, 178]. While rapid diagnostic tests are available for NS1 antigen and IgM testing, they have lower sensitivity and specificity than laboratory‐based NS1/IgM assays [179].

Severe dengue was reported in 13.0% of cases, higher compared to less than 5% in the general population [180]. Well‐demonstrated risk factors for severe dengue progression include young and older age, secondary DENV infection, diabetes, and renal disease [181, 182]. However, the relationship between SOT and severe dengue incidence or mortality remains controversial in the literature. One cohort study reported higher severe dengue incidence and mortality [102], but another reported no remarkable differences [119]. Emerging evidence suggests that an aggressive immune response to DENV infection can lead to severe disease, characterized by vascular leakage and excessive inflammation driven by high levels of inflammatory cytokines [183]. A large pediatric cohort study found that pre‐existing anti‐dengue antibodies significantly increase the risk of severe dengue, aligning with the theory of antibody‐dependent enhancement [184]. However, both adaptive and innate immune responses prevent viral replication, complicating the understanding of IS's effects on DENV infection. Notably, persistent dengue infection has been reported in SOT recipients [138, 185].

Current treatment approaches for DENV infection include supportive care with intravenous fluids, and transfusions if needed. We found that many cases of DENV among SOTRs involved reducing IS (30.1%). In our review, six SOTRs were treated with IVIG, with no reported deaths among these cases. Although a randomized controlled trial did not identify IVIG with clinical efficacy in the recovery of platelet counts, it may theoretically prevent vascular leak due to its anti‐inflammatory effects [186, 187]. One retrospective observational study suggested that IVIG could be used as a rescue therapy in severe refractory dengue [188]. However, randomized control studies studying the benefit of IVIG in DENV are lacking. Attributable mortality was 4.9%, also higher compared to less than 1% in the general population [189].

We also included less frequently reported ABF infections among SOTRs: JEV, POWV, SLEV, TBEV, YFV, and ZIKV. The most common epidemiological risk factor for these flavivirus infections included residence or travel to endemic areas for flavivirus infection [7]. While cases of donor‐derived WNV have been reported and previously discussed, we found several cases of donor‐derived TBEV and YFVV infections [151, 154]. Interestingly, the case series of donor‐derived YFVV infection occurred in the setting of an organ donor who received a blood transfusion from a person with recent YFV vaccination and active viremia, subsequently transmitting YFVV to multiple organ recipients [154]. YFV vaccination has previously been reported among SOTRs without increased risk of adverse effects [190, 191]. However, as SOTRs are not currently recommended to receive YFV vaccination, more data is needed on the prevalence and clinical manifestations of YFVV infection [8, 192]. The reported cases underscore the risk of YFV vaccination and transmission of YFVV through blood and organ donations, highlighting the need to defer blood donations for at least 2 weeks post‐vaccination. Furthermore, we identified various cases of transfusion‐derived JEV, POWV, SLEV, and ZIKV infections [144, 146–148, 156]. Bloodborne transmission of flaviviruses, including DENV, ZIKV, TBEV, JEV, POWV, and SLEV, has been well documented in the literature [193, 194]. As climate change is expected to increase the incidence of flavivirus infections, blood banks may need to implement screening for flaviviruses to prevent transfusion‐associated infections [7]. Regarding treatment, several cases reported the administration of IVIG [145, 148, 154]. Prior literature on cases with JEV, TBEV, and YFVV have included treatment with IVIG [195, 196, 197, 198, 199]. One case of SLEV in a SOTR also involved treatment with IFN [148]. Interestingly, some prior literature has shown a potential benefit from IFN therapy for infections caused by SLEV [200, 201, 202]. Unfortunately, several cases of ABF infections in SOTRs resulted in death, further highlighting the vulnerability of this population [8, 148, 151, 152, 154].

Our review found that SOTRs are vulnerable to severe presentations of ABF infections, highlighting the importance of the prevention of arthropod‐borne infections. Wearing permethrin‐treated protective clothing (particularly long‐sleeve shirts and pants) and effective insect repellents that contain DEET can be used by SOTRs to prevent both mosquito and tick‐borne infections [203]. To prevent WNV and other mosquito‐borne infections, SOTRs should also avoid going outdoor activities during peak mosquito feeding (at dawn or dusk [204]). Furthermore, to reduce the burden of mosquitos around patients’ homes, sources that promote standing water (such as old tires) should be removed [203]. To prevent tick‐borne infections, SOTRs should be exposed to wooded and brushy areas with high grass and leaf litter [204]. Notably, there are two WHO‐approved DENV vaccines: Dengvaxia and Qdenga (TAK‐003) [176, 205]. Dengvaxia is recommended only for individuals 6 through 16 years of age with previous laboratory‐confirmed DENV infection and living in endemic areas [176]. Qdenga is contraindicated for patients with acquired immune deficiency or those receiving immunosuppressives [205]. Thus, this vaccine is not recommended for adult SOTRs. Non‐live virus vaccination against JEV should be considered for SOTRs traveling to endemic regions, particularly during peak transmission season [206]. YFV vaccination contains a live attenuated strain. Transplant candidates living in or intending to travel to YFV endemic regions should be vaccinated at least 4 weeks before transplantation. Unvaccinated SOTRs should avoid travel to YFV‐endemic regions during its peak season [192]. Patients should be educated on mosquito and tick bite prevention measures prior to SOT and seek travel medicine consultation if traveling abroad to regions endemic to ABFs [203, 207].

Our study has several inherent limitations, primarily due to the scoping review nature of our study and the reliance on data from reported literature. We included abstracts from global conferences that did not consistently report all clinical features of infection. Since we pooled available data, no control groups were available for comparison with general or immunocompetent patients. Many cases occurred within 1 year post‐transplantation, which may be biased due to the short duration of follow‐up in many studies. Additionally, severe cases of ABF infections were more likely to be reported due to publication bias. We employed a systematic approach to our literature review to decrease the risk of bias. There was a notable difference in sex distribution, with males reported more frequently than females. Most cases of DENV reported infection among kidney transplant recipients (95.6%). Regarding maintenance IS, studies did not distinguish between CNIs, such as CsA and tacrolimus. This may be important to note as some studies suggest the beneficial effect of CsA on flavivirus infections [208, 209]. Furthermore, we did not specify the timing of positive serological, antigen, or molecular testing as many studies did not explicitly report the timing of tests. Reduction in IS was not clearly distinguished between discontinuation, dose reduction, or switching immunosuppressants, making it difficult to assess the impact of reducing IS on patient outcomes. While our review provides valuable insights into the impact of flavivirus infections in SOTRs, these limitations highlight the need for more robust and comprehensive studies with control groups, longer follow‐up periods, and detailed reporting of all relevant clinical factors.

## 5 Conclusion

According to reported literature, the most common ABF infections among SOTRs were WNV and DENV. Most ABF infections occur among patients living in endemic areas. DDI infections were reported for WNV, DENV, TBEV, and YFVV, and transfusion‐transmitted infections for WNV, JEV, POWV, SLEV, and ZIKV. Treatment for all ABF infections included supportive care and reduction in IS. For WNV, we found high rates of WNND, high mortality (23.7%), and a significant prevalence of severe neurological sequelae (43.9%). While for DENV, severe dengue was reported in 13.0% of cases, higher than the general population rate of 5%. ABF infections contributed to high morbidity and mortality for SOTRs, underscoring the need for novel approaches to disease prevention, early diagnostic testing, and treatment approaches.

## Supporting information



Supporting Information

Visual Abstract

## Data Availability

Research data are not shared.
