# Supplementary material for: A Scoping Review of Arthropod‐Borne Flavivirus Infections in Solid Organ Transplant Recipients
Source: Transpl Infect Dis. 2024 Nov 4;26(6):e14400. doi: 10.1111/tid.14400 (PMC11666879; doi:10.1111/tid.14400)
Supplement: Supplementary file 1 — Supporting Information [file TID-26-e14400-s002.docx]

**Supplementary Appendix**

**[Supplementary 1] Search strategies**

To identify studies for this scoping review, the review team collaborated with a medical librarian to develop detailed search strategies for each database. The PRISMA-S extension was followed for search reporting. The medical librarian developed the search for PubMed (National Library of Medicine; NLM) and translated it for each database searched. The PubMed (NLM) search strategy was reviewed by the research team for accuracy and relevancy of terms, and all final searches were peer-reviewed by another medical librarian following the PRESS checklist^1^. The databases included in this search are PubMed, Embase (embase.com), Cochrane CENTRAL Register of Controlled Trials (Wiley), and Global Index Medicus (World Health Organization; WHO), which were searched using a combination of keywords and subject headings. There were no search limits. Full searches were performed on January 25, 2024. The full search keywords are provided below.

PubMed (NLM)

| (flavivirus*[tiab] OR flaviviridae[tiab] OR dengue[tiab] OR DENV[tiab] OR “breakbone fever”[tiab] OR “break bone fever”[tiab] OR “Japanese encephalitis”[tiab] OR “Japanese B encephalitis”[tiab] OR “Japanese B viral encephalitis”[tiab] OR “Saint Louis encephalitis”[tiab] OR “St. Louis encephalitis”[tiab] OR “tickborne encephalitis”[tiab] OR “tick borne encephalitis”[tiab] OR Powassan[tiab] OR “Russian spring summer encephalitis”[tiab] OR “Russian encephalitis”[tiab] OR “Central European encephalitis”[tiab] OR “West Siberian virus”[tiab] OR “West Nile”[tiab] OR WNV[tiab] OR “Kunjin virus”[tiab] OR “yellow fever”[tiab] OR zika[tiab] OR ZIKV[tiab] OR “Usutu virus”[tiab] OR “Omsk hemorrhagic fever”[tiab] OR “Kyasanur forest disease”[tiab] OR “Alkhurma hemorrhagic fever”[tiab] OR “Murray Valley encephalitis”[tiab] OR “flavivirus infections”[mesh:noexp] OR dengue[mesh] OR “encephalitis, Japanese”[mesh] OR “encephalitis, St. Louis”[mesh] OR “encephalitis, tick-borne”[mesh] OR “hemorrhagic fever, Omsk”[mesh] OR “Kyasanur Forest Disease”[mesh] OR “West Nile fever”[mesh] OR “yellow fever”[mesh] OR “zika virus infection”[mesh] OR flavivirus[mesh]) AND (“solid organ transplant*”[tiab] OR “liver transplant*”[tiab] OR “hepatic transplant*”[tiab] OR “kidney transplant*”[tiab] OR “renal transplant*”[tiab] OR “heart transplant*”[tiab] OR “cardiac transplant*”[tiab] OR “lung transplant*”[tiab] OR “pulmonary transplant*”[tiab] OR “heart-lung transplant*”[tiab] OR “pancreas transplant*”[tiab] OR “pancreatic transplant*”[tiab] OR “intestine transplant*”[tiab] OR “intestinal transplant*”[tiab] OR “small bowel transplant*”[tiab] OR “kidney-pancreas transplant*”[tiab] OR “pancreas-kidney transplant*”[tiab] OR “liver-kidney transplant*”[tiab] OR “kidney-liver transplant*”[tiab] OR “lung-kidney transplant*”[tiab] OR “kidney-lung transplant*”[tiab] OR “liver-intestine transplant*”[tiab] OR “intestine-liver transplant*”[tiab] OR “intestine-kidney transplant*”[tiab] OR “kidney-intestine transplant*”[tiab] OR “transplant recipient*”[tiab] OR “transplant recipients”[mesh] OR “heart transplantation”[mesh] OR “heart-lung transplantation”[mesh] OR “kidney transplantation”[mesh] OR “liver transplantation”[mesh] OR “lung transplantation”[mesh] OR “pancreas transplantation”[mesh] OR “organ transplantation”[mesh:noexp]) |
| --- |

Embase (Elsevier, embase.com)

| #1 | (flavivirus*:ab,ti OR Flaviviridae:ab,ti OR dengue:ab,ti OR DENV:ab,ti OR ‘breakbone fever’:ab,ti OR ‘break bone fever’:ab,ti OR ‘Japanese encephalitis’:ab,ti OR ‘Japanese B encephalitis’:ab,ti OR ‘Japanese B viral encephalitis’:ab,ti OR ‘Saint Louis encephalitis’:ab,ti OR ‘St. Louis encephalitis’:ab,ti OR ‘tickborne encephalitis’:ab,ti OR ‘tick borne encephalitis’:ab,ti OR Powassan:ab,ti OR ‘Russian spring summer encephalitis’:ab,ti OR ‘Russian encephalitis’:ab,ti OR ‘Central European encephalitis’:ab,ti OR ‘West Siberian virus’:ab,ti OR ‘West Nile’:ab,ti OR WNV:ab,ti OR ‘Kunjin virus’:ab,ti OR ‘yellow fever’:ab,ti OR zika:ab,ti OR ZIKV:ab,ti OR ‘Usutu virus’:ab,ti OR ‘Omsk hemorrhagic fever’:ab,ti OR ‘Kyasanur Forest disease’:ab,ti OR ‘Alkhurma hemorrhagic fever’:ab,ti OR ‘Murray Valley encephalitis’:ab,ti OR ‘flavivirus infection’/de OR dengue/exp OR ‘Japanese encephalitis’/exp OR ‘Kyasanur Forest disease’/exp OR ‘Murray Valley encephalitis’/exp OR ‘Omsk hemorrhagic fever’/exp OR ‘St. Louis encephalitis’/exp OR ‘tick borne encephalitis’/de OR ‘Central European encephalitis’/exp OR ‘Powassan encephalitis’/exp OR ‘Russian spring summer encephalitis’/exp OR ‘West Nile fever’/exp OR ‘yellow fever’/exp OR ‘zika fever’/exp OR flavivirus/de OR ‘dengue virus’/exp OR ‘Japanese encephalitis virus group’/exp OR “Kyasanur Forest disease virus’/exp OR ‘tick borne flavivirus’/de OR ‘Powassan virus’/exp OR ‘tick borne encephalitis virus’/exp OR ‘yellow fever virus’/exp OR ‘zika virus’/exp) |
| --- | --- |
| #2 | (‘solid organ transplant*’:ab,ti OR ‘liver transplant*’:ab,ti OR ‘hepatic transplant*’:ab,ti OR ‘kidney transplant*’:ab,ti OR ‘renal transplant*’:ab,ti OR ‘heart transplant*’:ab,ti OR ‘cardiac transplant*’:ab,ti OR ‘lung transplant*’:ab,ti OR ‘pulmonary transplant*’:ab,ti OR ‘heart-lung transplant*’:ab,ti OR ‘pancreas transplant*’:ab,ti OR ‘pancreatic transplant*’:ab,ti OR ‘intestine transplant*’:ab,ti OR ‘intestinal transplant*’:ab,ti OR ‘small bowel transplant*’:ab,ti OR ‘kidney-pancreas transplant*’:ab,ti OR ‘pancreas-kidney transplant*’:ab,ti OR ‘liver-kidney transplant*’:ab,ti OR ‘kidney-liver transplant*’:ab,ti OR ‘lung-kidney transplant*’:ab,ti OR ‘kidney-lung transplant*’:ab,ti OR ‘liver-intestine transplant*’:ab,ti OR ‘intestine-liver transplant*’:ab,ti OR ‘intestine-kidney transplant*’:ab,ti OR ‘kidney-intestine transplant*’:ab,ti OR ‘transplant recipient*’:ab,ti OR ‘organ transplantation’/de OR ‘heart transplantation’/exp OR ‘intestine transplantation’/exp OR ‘kidney transplantation’/exp OR ‘liver transplantation’/exp OR ‘lung transplantation’/exp OR ‘pancreas transplantation’/exp OR ‘graft recipient’/exp) |
| #3 | #1 AND #2 |

Cochrane CENTRAL Register of Controlled Trials (Wiley)

| #1 | (flavivirus* OR flaviviridae OR dengue OR DENV OR “breakbone fever” OR “break bone fever” OR “Japanese encephalitis” OR “Japanese B encephalitis” OR “Japanese B viral encephalitis” OR “Saint Louis encephalitis” OR “St. Louis encephalitis” OR “tickborne encephalitis” OR “tick borne encephalitis” OR Powassan OR “Russian spring summer encephalitis” OR “Russian encephalitis” OR “Central European encephalitis” OR “West Siberian virus” OR “West Nile” OR WNV OR “Kunjin virus” OR “yellow fever” OR zika OR ZIKV OR “Usutu virus” OR “Omsk hemorrhagic fever” OR “Kyasanur forest disease” OR “Alkhurma hemorrhagic fever” OR “Murray Valley encephalitis”):ti,ab,kw |
| --- | --- |
| #2 | ((“solid organ” NEXT transplant*) OR (liver NEXT transplant*) OR (hepatic NEXT transplant*) OR (kidney NEXT transplant*) OR (renal NEXT transplant*) OR (heart NEXT transplant*) OR (cardiac NEXT transplant*) OR (lung NEXT transplant*) OR (pulmonary NEXT transplant*) OR (“heart-lung” NEXT transplant*) OR (pancreas NEXT transplant*) OR (pancreatic NEXT transplant*) OR (intestine NEXT transplant*) OR (intestinal NEXT transplant*) OR (“small bowel” NEXT transplant) OR (“kidney-pancreas” NEXT transplant*) OR (“pancreas-kidney” NEXT transplant*) OR (“liver-kidney” NEXT transplant*) OR (“kidney-liver” NEXT transplant*) OR (“lung-kidney” NEXT transplant*) OR (“kidney-lung” NEXT transplant*) OR (“liver-intestine” NEXT transplant*) OR (“intestine-liver” NEXT transplant*) OR (“intestine-kidney” NEXT transplant*) OR (“kidney-intestine” NEXT transplant*) OR (transplant NEXT recipient*)):ti,ab,kw |
| #3 | #1 AND #2 |

Global Index Medicus (World Health Organization)

| (tw:(flavivirus* OR dengue OR encephalitis OR "west nile" OR "yellow fever" OR zika)) AND (tw:(transplant OR transplants OR transplantation)) |
| --- |

**[Supplementary 2] Screening process and inclusion/exclusion criteria**

**1. Identification**

1217 studies were identified from the initial database and register search and imported to Covidence (<https://www.covidence.org/>). Covidence marked and omitted 286 duplicate studies, with an additional 6 duplicates identified by the librarian.

**2. Abstract and title screening**

925 studies were included for abstract and title screening. Two independent reviewers used Covidence to screen abstracts and titles for eligibility. In cases of disagreement, a third reviewer acted as a tiebreaker.

If the authors were the same, the research institution, published date, timeframe, and abstract contents (case descriptions) were compared. Non-overlapping studies were considered non-repetitive.

For overlapping studies, the following inclusion principles were applied:

- Select the article with the largest sample size.
- Select the article with the longest follow-up time.
- Choose the article with the most comprehensive research outcome.

Studies were excluded if they met the following criteria:

- Wrong study population: age < 18 years, hematopoietic stem cell transplantation recipient, transfusion recipient without a history of solid organ transplant (SOT), or donors only.
- Wrong study design: review article, meta-analysis, brief communication without case description, or guidelines.
- Wrong object: other than arthropod-borne flaviviruses (ABF) as listed in Table S1, ABF before SOT.

**3. Full text screening**

241 studies were selected for full-text screening. Full-text information about the research institution, timeframe, and study object was reviewed. Studies from the same country and research center were compared to ensure non-overlapping research subjects or timeframes. The same rules as abstract and title screening were used for overlapping studies and reviewers with a tiebreaker.

- For example, Patel 2020^2^ is an abstract about a prospective observational study on Dengue virus (DENV) infection in renal transplant recipients from 2018 to 2019 at IKDRC-ITS, Ahmedabad, India. Meshram 2021^3^ is also an observational study from July 2019 to April 2020 at the same institution with the same number of DENV cases (n=31). Due to possible overlap, the less comprehensive study (Patel 2020) was excluded.
- Another example is the Morbidity and Mortality Weekly Report (MMWR) 2002^4^, which documented West Nile virus (WNV) infection cases, overlapping with Jain 2007^5^ but included one non-overlapping case. Both studies were included for data extraction, but data from overlapping cases were taken from Jain 2007, as it had more detailed case descriptions.

Additional exclusion criteria for full-text screening included:

- Unable to find the full text.
- Published in a language other than English, Spanish, or Portuguese.
- Wrong study subject: serologic study, immunization prevalence, or in-vitro only study.

**[Supplementary 3] Data extraction strategies and definitions**

146 studies were included in the data extraction process. References were exported from Covidence to an Excel spreadsheet, with each column representing a data entry. Discrepancies regarding study eligibility or data extraction were resolved by consensus and final decision by Foppiano Palacios C. The following information was collected from each study: baseline characteristics, predisposing factors, clinical characteristics, and treatment course/outcome. The defined criteria are described below.

**1. Baseline Characteristics:**

- Ages were recorded exactly from case reports or series. If an age range was given (e.g., "seventies"), the midpoint (75) was used for mean age calculations. Mean age with standard deviation was also included.
- Regions for WNV cases were categorized as North America, Europe, Middle East, South/Central America, Asia, Africa, Oceania, and others. For DENV cases, WHO regional classifications were used: South-east Asia, South America, Western-Pacific, East-Mediterranean, and Africa.
- Transplanted organ information was categorized as: kidney, liver, heart, lung, intestine, pancreas, kidney/pancreas, and others. Donor status (deceased or living) was noted if reported.

**2. Predisposing factors**

- Human immunodeficiency virus infection and diabetes mellitus were recorded if reported.
- Endemicity exposure was determined by the location of the institution and travel history. Exposure was attributed to residence if the case was from an endemic area with no travel history, or to travel if the case was from a non-endemic area with a travel history to an endemic area. If the case was from an endemic area with travel history to endemic area as well, it was attributed to residence.
- Induction therapy was extracted and included anti-thymocyte globulin (ATG), basiliximab, alemtuzumab, and muromonab. For exceptions, such as in Ng 2019^6^, ATG use was counted due to its addition for delayed graft function between thje second and eleventh days post-SOT.
- Maintenance immunosuppression (IS) therapy at infection onset included calcineurin inhibitors, mycophenolate, steroids, azathioprine, and sirolimus. Rejection history prior to infection was recorded, with specific identification if it occurred within 6 months or 1 year before the onset of infection.

**3. Clinical characteristics**

- Symptom onset post-SOT was categorized by intervals: <30 days, 30 days to 1 year, >1 year. Asymptomatic cases recorded the date of the first positive diagnostic test. Transmission from donor or transfusion was noted as reported.
- Presentations from onset through progression during admission were included. Presentations for WNV included fever, altered mental status, weakness/paralysis, headache, diarrhea, seizures, neck stiffness, or asymptomatic. For DENV, presentations included fever, myalgia, headache/retro-orbital pain, nausea/vomiting, arthralgia, diarrhea, bleed/hemorrhage, abdominal pain, rash, or asymptomatic.
- Diagnostic methods for WNV included serum IgM, serum IgG, plaque reduction neutralization tests, serum PCR, cerebrospinal (CSF) IgM, CSF IgG, and CSF PCR if positive post-infection. CSF studies noted lymphocytic pleocytosis (WBC > 5/µL, lymphocytes >50%) and elevated protein (>45 mg/dL). Autopsy and brain biopsy results were counted if diagnostic. MRI and CT head reports were collected if available.
- Lab findings in DENV included thrombocytopenia (platelets <150,000/µL), leukopenia (WBC <4,000/µL), elevated aspartate aminotransferase (AST) or alanine aminotransferase (ALT) levels (AST or ALT > 80 U/L; roughly 2 times the upper limit of normal), anemia (Hemoglobin (Hb) <13 g/dL for men, <12 g/dL for women), neutropenia (absolute neutrophil count <1,500/µL), and lymphopenia (absolute lymphocyte count <1,500/µL).
- Severe dengue was recorded if defined as "severe dengue" or if symptoms/signs aligned with WHO 2009 guidelines; shock (including “dengue shock syndrome,” “shock,” or hypotension needing vasopressors); severe bleeding (“severe bleeding,” acute bleeding needing red blood cell transfusion, or Hb <7); respiratory distress due to fluid accumulation or needing advanced support (intubation or non-invasive ventilation); AST or ALT ≥1000 U/L; and other severe organ involvements.
- Diagnostic method for DENV: NS1 Antigen, serum IgM, serum IgG, serum PCR, and others.

**4. Treatment course and outcome**

- Targeted treatments were noted for WNV and DENV, were noted, excluding empirical antivirals like acyclovir. IS reduction was recorded if decreased, discontinued, or switched. Graft loss and post-infection rejection were collected. Deaths were categorized as infection-related if reported as such, or if the patient died during hospitalization due to severe ABF infection without another cause.
- For WNV sequelae, severe neurological complication was defined if: patient ended up with coma, paralysis, discharge to long-term acute care, or non-specified but described as “severe neurologic damage” or multiple neurological complications requiring rehabilitation.

1. McGowan J, Sampson M, Salzwedel DM, Cogo E, Foerster V, Lefebvre C. PRESS Peer Review of Electronic Search Strategies: 2015 Guideline Statement. *J Clin Epidemiol*. 2016;75:40-46. doi:10.1016/j.jclinepi.2016.01.021

2. Patel H, Kute V, Banerjee S, Engineer D. CHANGING SEVERITY OF DENGUE INFECTION IN RENAL TRANSPLANT RECIPIENTS: “UNPREDICTABLE AND LETHAL.” *Transplantation*. 2020;104(S3):S371-S371. doi:10.1097/01.tp.0000700436.55299.3a

3. Meshram HS, Kute V, Patel H, Banerjee S, Chauhan S, Desai S. Successful management of dengue in renal transplant recipients: A retrospective cohort from a single center. *Clin Transplant*. 2021;35(7):e14332. doi:10.1111/ctr.14332

4. Update: Investigations of West Nile virus infections in recipients of organ transplantation and blood transfusion. *MMWR Morb Mortal Wkly Rep*. 2002;51(37):833-836.

5. Jain N, Fisk D, Sotir M, Kehl KS. West Nile encephalitis, status epilepticus and West Nile pneumonia in a renal transplant patient. *Transpl Int*. 2007;20(9):800-803. doi:10.1111/j.1432-2277.2007.00514.x

6. Ng KH, Zhang SL, Tan HC, et al. Persistent Dengue Infection in an Immunosuppressed Patient Reveals the Roles of Humoral and Cellular Immune Responses in Virus Clearance. *Cell Host Microbe*. 2019;26(5):601-605.e3. doi:10.1016/j.chom.2019.10.005
