# Supplementary material for: A Scoping Review of Arthropod‐Borne Flavivirus Infections in Solid Organ Transplant Recipients
Source: Transpl Infect Dis. 2024 Nov 4;26(6):e14400. doi: 10.1111/tid.14400 (PMC11666879; doi:10.1111/tid.14400)
Supplement: Supplementary file 2 — Visual Abstract [file TID-26-e14400-s001.pptx]

## Slide 1
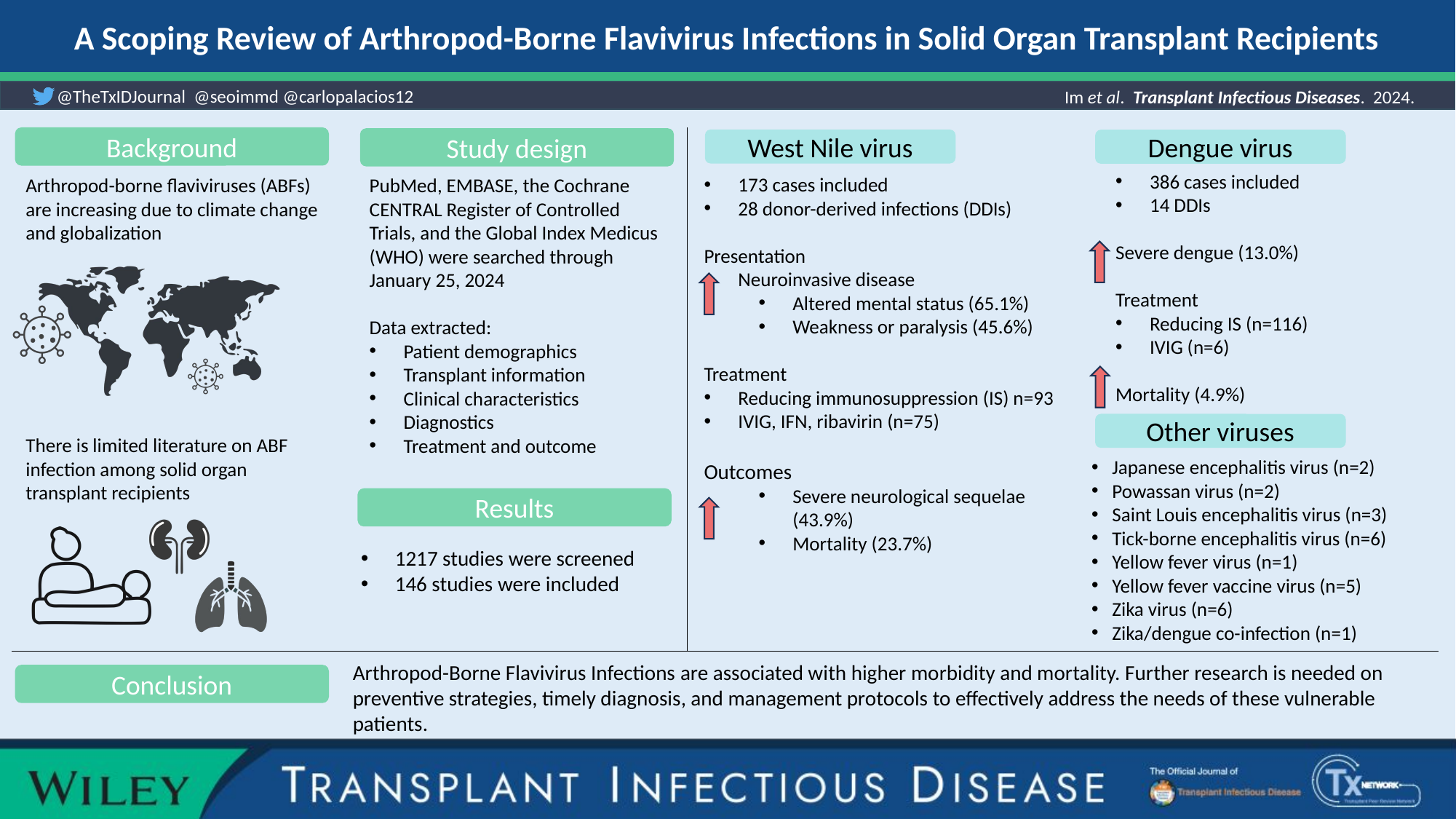

A Scoping Review of Arthropod-Borne Flavivirus Infections in Solid Organ Transplant Recipients
Im et al. Transplant Infectious Diseases. 2024.
 @TheTxIDJournal @seoimmd @carlopalacios12
Background
Study design
West Nile virus
Dengue virus
386 cases included
14 DDIs
Severe dengue (13.0%)
Treatment
Reducing IS (n=116)
IVIG (n=6)
Mortality (4.9%)
173 cases included
28 donor-derived infections (DDIs)
Presentation
Neuroinvasive disease
Altered mental status (65.1%)
Weakness or paralysis (45.6%)
Treatment
Reducing immunosuppression (IS) n=93
IVIG, IFN, ribavirin (n=75)
Outcomes
Severe neurological sequelae (43.9%)
Mortality (23.7%)
Arthropod-borne flaviviruses (ABFs) are increasing due to climate change and globalization
There is limited literature on ABF infection among solid organ transplant recipients
PubMed, EMBASE, the Cochrane CENTRAL Register of Controlled Trials, and the Global Index Medicus (WHO) were searched through January 25, 2024
Data extracted:
Patient demographics
Transplant information
Clinical characteristics
Diagnostics
Treatment and outcome
Other viruses
Japanese encephalitis virus (n=2)
Powassan virus (n=2)
Saint Louis encephalitis virus (n=3)
Tick-borne encephalitis virus (n=6)
Yellow fever virus (n=1)
Yellow fever vaccine virus (n=5)
Zika virus (n=6)
Zika/dengue co-infection (n=1)
Results
1217 studies were screened
146 studies were included
Arthropod-Borne Flavivirus Infections are associated with higher morbidity and mortality. Further research is needed on preventive strategies, timely diagnosis, and management protocols to effectively address the needs of these vulnerable patients.
Conclusion
